# Dietary fatty acids and incident hip fractures in cohorts of women and men. A relative validation and follow-up study

**DOI:** 10.1016/j.jnha.2024.100247

**Published:** 2024-04-25

**Authors:** Eva Warensjö Lemming, Liisa Byberg, Jonas Höijer, Susanna C. Larsson, Alicja Wolk, Karl Michaëlsson

**Affiliations:** a Medical Epidemiology, Department of Surgical Sciences, Uppsala University, Uppsala, Sweden; b Department of Food Science, Nutrition and Dietetics, Uppsala University, Uppsala, Sweden; c Unit of Cardiovascular and Nutritional Epidemiology, Institute of Environmental Medicine, Karolinska Institutet, Stockholm, Sweden

**Keywords:** Cohort study, Biomarker fatty acids, Hip fractures, Diet, Validation, Nutrition

## Abstract

**Objectives:**

Hip fractures are associated with a high burden of morbidity and mortality. Diet is essential for preventing fragility fractures, but the role of dietary fatty acids on the risk of hip fracture is uncertain. The aim was to investigate how intake of different dietary fatty acids relates to the risk of hip fracture. A relative validation of the long-term intake of dietary fatty acids estimated from food frequency questionnaires (FFQs) was also performed.

**Design, settings and participants:**

We used data collected in two population-based cohorts, the Swedish Mammography Cohort and the Cohort of Swedish men (n = 83,603, 54% men, aged 45–82 years). Data from the repeated investigations in the cohorts and cross-sectional data from their clinical sub-cohorts were used.

**Measurements:**

Diet data was collected in FFQs. Incident hip fractures were gathered by individual linkage to national registers. We performed Cox regression analysis to investigate associations between dietary fatty acids and hip fracture. Follow-up time was between January 1st, 1998 and December 31st, 2020. The validation was performed using correlation analyses, comparing fatty acids measured in adipose tissue with estimated fatty acid intakes from FFQs.

**Results:**

During up to 23 years of follow-up (mean 18 years) and 1,538,627 person-years at risk, 7345 participants (2840 men) experienced a hip fracture. A low linoleic acid (LA) and high intakes of long-chain n-3 fatty acids were associated with higher hip fracture risk in a non-linear way. In quartile 4 compared to quartile 1 of LA, the multivariable-adjusted hazard ratio of hip fracture was 0.89 (95% Confidence Interval: 0.81, 0.97). The study confirmed the validity of FFQs to capture the intake of the specific dietary long-chain n-3 fatty acids. The estimated intake of LA, α-linolenic acid, and myristic acid were also adequately captured by the FFQs. Validity was confirmed in both women and men.

**Conclusion:**

A low to moderate intake of linoleic acid and a higher intake of long-chain n-3 fatty acids were associated with a higher risk of hip fractures. The results indicate that attention should be paid to dietary fatty acid composition for the optimal prevention of fragility fractures.

## 1 Introduction

Hip fractures are associated with a high burden of morbidity and mortality and increasing healthcare costs due to ageing populations; yearly, millions worldwide suffer from fragility fractures [[1], [2], [3]]. The importance of a healthy diet for preventing fragility fractures has been emphasized [4]. Many nutrients, including dietary fatty acids and other bioactive components, may be involved in maintaining bone health [5,6]. From a preventive and health promotion perspective, it is advised to primarily use vegetable oils or mixed fat blends in food preparation and to choose dairy products with lower fat content. In doing so, the dietary content of unsaturated fatty acids increases at the expense of saturated fatty acids while ensuring the intake of the essential fatty acids [7] known to associate with beneficial health effects [8]. It is known that certain fatty acids exert cellular effects that may decrease bone resorption by osteoclasts while stimulating bone formation by osteoblasts, while other fatty acids have opposite effects [9]. In addition, polyunsaturated fatty acids (PUFA) may improve muscular function [10]. The risk of fracture is not only related to age-related bone loss [11] but also to sarcopenia [12,13], which influences balance and falls. Sarcopenia and age-related bone loss share common pathological processes, such as inflammation [10,14].

Previous studies on the association between dietary fat in general and specific dietary fatty acids in particular and the risk of hip fracture have produced conflicting results. A narrative review 2013 reported a modest direct association between PUFA and bone mineral density (BMD) in older adults, possibly translating to a reduced fracture risk [9]. Another study suggested that saturated fatty acid intake increased, while unsaturated fatty acid intake decreased total fracture risk [15], findings not supported by the study by Virtanen et al. [16]. More recently, two systematic reviews and meta-analyses have proposed that total dietary n-3 fatty acids may relate inversely to hip fracture risk [17], that total dietary fat has no association, and that dietary saturated fatty acids are positively associated with hip fracture risk [18]. Studies of individual fatty acids in relation to fracture risk is mostly lacking and thus the relationship between individual dietary fatty acids and hip fracture risk warrants further investigation.

Selective underreporting of foods associated with a negative health image will impact the estimated intake of dietary fatty acids [19], potentially leading to misclassification bias. Interpretation of results using diet data is therefore dependent on the ability of the dietary assessment method to capture intake of dietary fatty acids accurately. Biomarker fatty acids, measured in plasma, erythrocyte and platelet membranes, and adipose tissue, partly mirror the intake of dietary fatty acids. Especially the essential fatty acids, linoleic acid (LA, 18:2 n-6) and α-linolenic acid (ALA, 18:3 n-3) and long-chain n-3 fatty acids (eicosapentaenoic acid (EPA, 20:5 n-3), docosapentaenoic acid (DPA, 22:5 n-3) and docosahexaenoic acid (DHA, 22:6 n-3). may mirror dietary intake [20]. Endogenous fatty acid metabolism [21] also influences biomarker fatty acid compositions. However, it is possible to use biomarker fatty acids to investigate the validity of dietary fatty acid intake estimated by a food frequency questionnaire (FFQ) [22]. One modestly large case-control study showed that older patients with hip fractures had lower plasma concentrations of total, n-3, and n-6 polyunsaturated fatty acids [23], suggesting that certain fatty acids may be associated with hip fracture risk.

Using dietary information collected in two large population-based studies, Swedish Mammography Cohort (SMC) and Cohort of Swedish Men (COSM), and register-based hip fracture events, the present study aims to investigate the estimated long-term intake of individual dietary fatty acids and hip fractures risk. We further use fatty acid biomarkers measured in adipose tissue in a subgroup of the cohort participants to perform a relative validation of the ability of the FFQs to capture the estimated long-term fatty acid intakes.

## 2 Methods

The study population comprises participants from two cohort studies and their respective sub-cohorts. The primary analysis was the prospective study investigating the FFQ-based dietary fatty acids intake and hip fracture risk in women and men. A pooled analysis was also enabled by using identical FFQs in women and men. The second analysis investigated the ability of the FFQs to capture long-term dietary fatty acid intake in the sub-cohorts. Notably, only the dietary fatty acids best captured by the FFQs were used in the prospective analyses.

### 2.1 Study cohorts

**Fig. 1:**
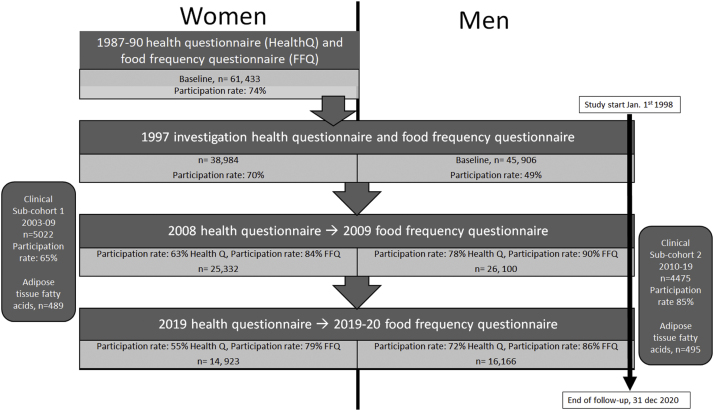
Flow-chart of the participation in the different investigations in the Swedish Mammography Cohort (Women) and Cohort of Swedish Men (Men) and the sub-cohorts. The flow-chart includes the year of the investigation, the number of participants, and participation rates. The number of adipose tissue fatty acids available in the sub-cohorts is also depicted. Health questionnaire (HealthQ), Food frequency questionnaire (FFQ).

Participants in the present study partook in two large population-based cohort studies: The SMC and COSM in Sweden, which are part of the national research infrastructure: Swedish Infrastructure for Medical Population-Based Life-Course and Environmental Research SIMPLER, (http://www.simpler4health.se/). The participants have partaken in repeated diet, lifestyle, and health assessments, and a detailed description of the study populations and assessments has been presented previously [24]. Fig. 1 outlines the different investigations in the cohorts and their respective sub-cohorts, including information on the number of participants and participation rates. SMC was established in 1987–1990, and a second investigation in SMC occurred in 1997, at the same time as COSM was initiated. The 1997 questionnaire used in both cohorts was similar. It included almost 350 items on diet (FFQ) and other lifestyle factors, for example, socio-demographic data, weight, height, total physical activity, self-perceived health status, smoking status, alcohol consumption, and use of dietary supplements. Re-investigations were further performed in 2008/2009 and 2019. Subjects were excluded from the analytical sample if the national registration number was missing, the questionnaire had not been dated, or energy intakes were deemed implausible (± 3 SD from the mean value of the log-transformed energy intake) at each follow-up. The study sample comprised 83,603 participants from the baseline questionnaire examination in late 1997, with a follow-up through 2020.

Data from the two clinical sub-cohorts of the SMC and COSM were used for the relative validation study (Fig. 1, Flow-chart). Female participants for sub-cohort 1 were recruited between 2003 and 2009 as a random sample of SMC participants living in Uppsala, and male participants for sub-cohort 2 were recruited from COSM participants between 2010 and 2019. Participants filled in questionnaires, including FFQ, and underwent health examinations. More information regarding the sub-cohorts was previously described in more detail [25,26]. Adipose tissue samples used for fatty acid analysis were collected at the health examination from the first 500 participants of each sex. The collection procedure has previously been described [25]. This study is reported adhering to the guideline, the Strengthening the Reporting of Observational Studies in Epidemiology- Nutritional Epidemiology (STROBE-nut) [27].

### 2.2 Measurements

#### 2.2.1 Dietary assessment

The dietary assessment has been described previously [28]. The FFQs included 67, 96, 132, and 132 food items in 1987, 1997, 2009, and 2019, respectively. Participants indicated in the FFQs how often, on average, they had consumed each food item during the last year and chose from eight predefined frequency categories ranging from "never/seldom" to "3 or more times per day". Frequently consumed foods such as dairy products and bread were additionally reported as number of servings per day. Information on fat type used in cooking and as salad dressing was also reported. The total amount of alcohol consumed daily was derived from the FFQ by multiplying the reported frequency with the reported amount on a single occasion. Energy and nutrient intakes were estimated by multiplying the consumption frequency of each food item with the nutrient content of sex and age-specific portion sizes. Nutrient values were obtained from the Swedish food composition database, Swedish Food Agency. Nutrient intakes were adjusted for total energy intake using the residual method [29]. Nutrient intakes excludes intake from supplements.

#### 2.2.2 Fatty acid analysis

Adipose tissue samples were taken from the upper buttocks, and the samples were protected from light and stored at −80 °C for a maximum of 18 months until analyzed. Fatty acids were extracted and transmethylated according to a previously described procedure [30]. The fatty acid methyl esters were separated by gas-liquid chromatography [25]. Fatty acids were identified by comparing each peak's retention time with fatty acid methyl ester standard Nu Check Prep (Elysian, MN, USA). The number of participants with analysed adipose tisse fatty acids and used in the validation analyses were 489 in sub-cohort 1 and 495 in sub-cohort 2.

#### 2.2.3 Exposures

The main exposures in the investigation of the hip fracture risk were the residual energy-adjusted intake of dietary fatty acids (grams per day), treated as quartiles and SD-scores in the linear models and as continuous variables in models exploring nonlinearity using cubic splines. In the clinical sub-cohorts, the individual fatty acid content of adipose tissue was treated as the relative fatty acid content (% of total fatty acids). This approach was also used in the validation analyses for the individual dietary fatty acids estimated from the FFQs, as described in the Statistical Analysis section below.

#### 2.2.4 Hip fractures

As outcomes, we considered incident hip fractures registered in the Swedish National Patient Registry [31] between study baseline 1 January 1998 and 31 December 2020, defined by the ICD10 codes S720, S721 and S722. Incident hip fracture events were distinguished from recurrent events using a previously validated and accurate method [32].

#### 2.2.5 Covariates

The covariates were chosen based on previous knowledge and using directed cyclic graphs. The information on covariates was obtained from the questionnaires and were the following: age, height, Body Mass Index (BMI) (all continuous), smoking status (current, former, never), living alone (binary), educational level (≤9, 10−12, >12 years, other), use of calcium and vitamin D supplements (binary), cortisone use (binary), walking/cycling (never/seldom, <20 min.d, 20−40 min/d, 40−60 min/d, 1−1.5 h/d, >1.5 h/d), leisure time physical exercise during the past year (<1 h/w, 1 h/w, 2−3 h/w, 4−5 h/w, >5 h/w). BMI was calculated as weight (kg) divided by height squared (m^2^). Comorbidity, expressed as Charlson’s weighted comorbidity index [33,34] was defined by ICD diagnosis codes (versions 8, 9 and 10) from the National Patient Registry. Total energy intake, intake of fruits and vegetables, alcohol, and intakes of dairy products (non-fermented milk, fermented milk, and cheese), fish, total meat, protein, vitamin D and calcium, all estimated from the FFQs, were also included as confounders (all continuous).

### 2.3 Statistical analysis

#### 2.3.1 Analyses of different dietary fatty acid intakes and hip fracture risk

As exposures in the prospective analyses of hip fracture risk, we only used the dietary fatty acids that were best captured by the FFQs in our relative validation study (see results below). Therefore, we used Cox proportional hazard regression analysis to investigate the association between dietary LA, ALA, EPA, DPA, DHA, and myristic acid and the risk of incident hip fractures. For each participant, follow-up time was accrued from baseline (1 January 1998) until the date of the first hip fracture, date of death, or the end of the study period (31 December 2020). All prospective analyses were done in the combined sample of both women and men and in each sex separately. All model variables, exposure, and covariates, except educational level and age, were updated at the time of the 2009 and 2019 investigations. This means that the participants who remain in the study after the re-investigations in 2009 and 2019 get an updated value for all model variables after each investigation. Fatty acids were modelled as quartiles with the first quartile as the reference, SD-scores (mean 0, SD = 1), and restricted cubic splines with three knots placed at the 10th, 50th, and 90th percentile, as Harrell recommended [35]. The reference level for the graphical representation of the models using the cubic splines was set to the median intake of respective fatty acid. We ran multivariable models, including covariates described above except dairy products, in women and men separately and in the pooled sample of all participants.

#### 2.3.2 Sensitivity analyses

In a sensitivity analyses we additionally included other dietary components as confounders. In the first sensitivity analysis, dairy products were added to the multivariable model. As a second sensitivity analysis, we added fish and total meat to the multivariable model and in a third sensitivity analysis protein, vitamin D and calcium were added. A fourth sensitivity analysis were performed and this included all covariates from the main analysis and sensitivity analyses 1−3. As a fifth sensitivity analysis we investigated the association between total intake of saturated fatty acids (SFA), and the individual saturated fatty acids lauric acid (C12:0), palmitic acid (C16:0) and stearic acid (C18:0) and multivariable adjusted risk of hip fracture.

Any missing data were multiple imputed using 20 imputations, taking into account model variables [36] and missing data were lower than 1% for most variables. Most missing data were for exercise (11%), followed by physical activity (9%), chicken intake (9%), living alone (6.5%), and BMI (4%).

#### 2.3.3 Relative validation of the fatty acid intake estimated by FFQ

The relative validation between fatty acid intake estimated from the different FFQs and adipose tissue fatty acids was carried out similarly to a previous comparative validation in sub-cohort 1 [22]. This earlier study established the ability of the FFQs to capture the intake of the long-chain n-3 fatty acids, EPA, DPA, and DHA using fewer participants (n = 239) of sub-cohort 1 [22]. Fatty acid content in adipose tissue (% of total fatty acids), and the relative dietary intake of fatty acids (% of total fat intake) estimated from the FFQs were used in the correlation analyses. We investigated the associations between the estimated intake of the following fatty acids: myristic acid (C14:0), palmitic acid (C16:0), stearic acid (C18:0), palmitoleic acid (C16:1), oleic acid (C18:1), LA, ALA, arachidonic acid (C20:4 n-6, AA), EPA, DPA and DHA and the corresponding fatty acids measured in adipose tissue. The intake of the individual dietary fatty acids among the participants in the sub-cohorts was estimated from different FFQ assessments in respective cohorts (SMC 1987, 1997 and sub-cohort 1 in women, and COSM 1997, 2009 and sub-cohort 2 in men). In participants of sub-cohort 1 used in the present validation analyses, the diet was assessed by two similar FFQs. Spearman and Pearson correlations with 95% confidence intervals (CIs), produced with bootstrapping over 1000 replications, were used to determine correlations between fatty acid intakes from the individual FFQs and the long-term average fatty acid intake (mean of all FFQs), and the adipose tissue fatty acid content. Partial correlation coefficients were established by adjusting for age at participation, BMI (kg/m^2^), energy intake, and physical activity level. In sub-cohort 1, information on recent physical activity levels was unavailable for all participants; thus, two models were applied: Model 1 excluded physical activity (n = 484), and Model 2 included physical activity level (n = 235).

All analyses were performed using Stata, version 15.1 (StataCorp, College Station, Texas, USA) on resources provided by the Swedish National Infrastructure for Computing’s (https://www.snic.se/) support for sensitive data (SNIC-SENS) through the Uppsala Multidisciplinary Center for Advanced Computational Science (UPPMAX) under Project SIMP2020010.

## 3 Results

### 3.1 Baseline characteristics

The baseline characteristics of the women and men in the 1997 investigation, used as a baseline in the prospective analysis, are shown in Table 1. The participants were around 60 years old (range 45–83 y) when the follow-up started, and the women were on average around two years older. The energy-adjusted intake of nutrients, including fatty acids, and the absolute intake of different food groups was numerically higher in men. The intake of the essential fatty acids LA and ALA was 20 times higher than that of the long-chain n-3 fatty acids (EPA, DPA, and DHA).

**Table 1:** Baseline characteristics among participants in the Swedish Mammography Cohort (SMC) and the Cohort of Swedish Men (COSM) at the 1997 investigation. Number of subjects (percentage) is shown if not otherwise indicated.

Characteristics		Women in SMC	Men in COSM
Number of subjects		38, 426	45, 906
Age (years), mean (SD)		62.5 (9.3)	60.8 (9.7)
Educational level	≤9 years	16050 (41.8)	15998 (35.0)
	10−12 years	15046 (39.2)	22281 (48.8)
	>12 years	7298 (19.0)	7415 (16.2)
Height (cm), mean (SD)		165 (5.8)	177 (6.7)
BMI (kg/m^2^), mean (SD)		25.1 (4.0)	25.8 (3.5)
Calcium supplement, any type	Yes	10011 (26.1)	6287 (13.7)
Calcium supplement	Yes	2555 (6.6)	911 (2.0)
Vitamin D supplement, any type	Yes	9105 (23.7)	7239 (15.8)
Cortisone use	Yes	5408 (14.1)	3024 (6.6)
Excercise	<1 h/week	6812 (19.8)	8982 (22.0)
	1 h/week	8101 (23.6)	7755 (19.0)
	2−3 h/week	11516 (33.6)	12793 (31.3)
	4−5 h/week	4061 (11.8)	5231 (12.8)
	>5 h/week	3834 (11.2)	6158 (15.0)
Walking	Never/seldom	3932 (11.1)	5672 (13.7)
	<20 min. day	6701 (18.9)	10045 (24.2)
	20−40 min/day	12104 (34.2)	12102 (29.2)
	40−60 min/day	6567 (18.6)	6340 (15.3)
	1−1.5 h/day	3613 (10.2)	3711 (8.9)
	>1.5 h/day	2453 (6.9)	3636 (8.8)
Smoking status	Current	8714 (23.1)	11293 (25.0)
	Former	8660 (23.0)	17568 (38.9)
	Never	20358 (54.0)	16357 (36.2)
Charlson’s comorbidity index	0	31932 (83.1)	37776 (82.3)
	1	3411 (8.9)	5390 (11.7)
	≥2	3083 (8.0)	2740 (6.0)
Living alone	Yes	8106 (24.4)	8048 (17.6)
Energy intake, kcal, mean (SD)		1735 (528)	2648 (912)
Mean (SD) daily intakes of residual adjusted nutrients			
Vitamin D, μg		4.5 (1.7)	6.7 (3.1)
Retinol, gram		0.9 (0.7)	1.3 (0.9)
Phosphorous, mg		1403 (230)	2070 (344)
Protein, gram		71 (12)	102 (15)
Calcium, mg		1043 (301)	1467 (471)
Alcohol, gram		4.1 (5.2)	10.3 (11)
Saturated fat, gram		28 (12)	42 (19)
Myristic acid (C14:0), gram		3.3 (1.1)	4.9 (1.6)
Linoleic acid (18:2 n-6), LA, gram		6.1 (1.6)	9.0 (2.1)
α-Linolenic acid (18:3 n-3), ALA, gram		1.2 (0.27)	1.7 (0.36)
Eicosapentaenoic acid (20:5 n-3), EPA, gram		0.10 (0.09)	0.16 (0.15)
Docosapentaenoic acid (22:5 n-3), DPA, gram		0.04 (0.02)	0.06 (0.05)
Docosahexaenoic acid (22:6 n-3), DHA, gram		0.22 (0.17)	0.32 (0.31)

Mean (SD) daily consumption of food groups (frequency per day)			
Cheese		2.7 (2.1)	3.0 (2.4)
Fermented milk		0.87 (1.0)	0.74 (1.0)
Milk		1.16 (1.3)	1.42 (1.5)
Meat		1.2 (0.8)	1.4 (0.9)
Fish and shellfish		0.5 (0.45)	0.5 (0.50)
Fruits and vegetables		5.1 (2.9)	3.9 (2.5)
Chicken		0.1 (0.1)	0.1 (0.1)

SD: standard deviation.

### 3.2 Dietary fatty acids and hip fracture risk

**Fig. 2:**
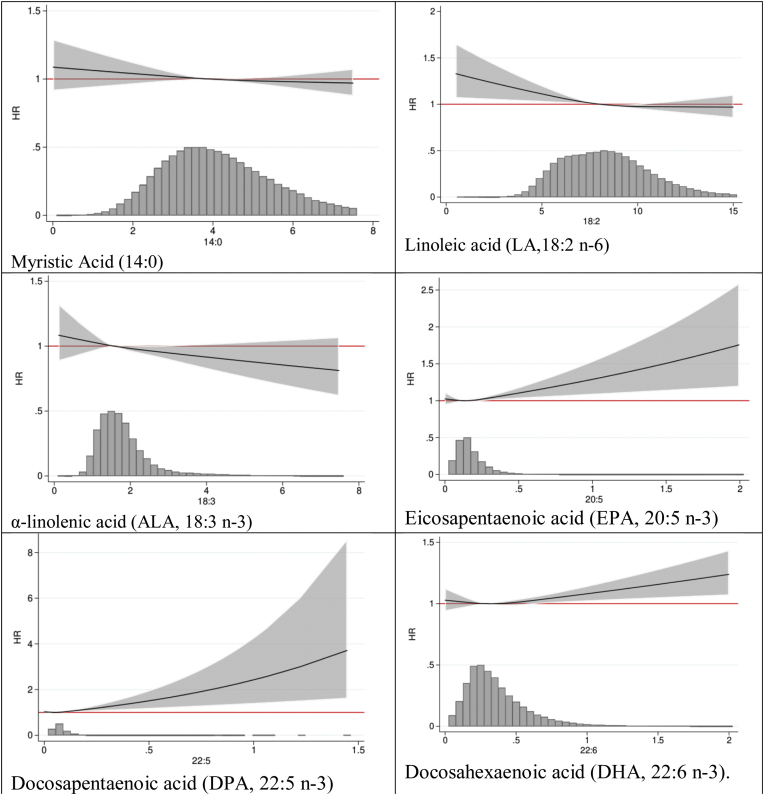
The figure shows the multivariable adjusted Hazard Ratio (HR, black line) and 95% Confidence Intervals (CI, shaded area) of hip fracture using restricted cubic-splines of time-updated dietary fatty acids in the overall pooled sample. The median intake level of respective fatty acid was used as the reference level in the figure. Depicted below the curve is the distribution of the dietary fatty acid intake among the participants. The intake levels are depicted as the residual adjusted intake in grams per day (x-axis). The multivariable model adjusted for age, height, Body Mass Index, smoking status, living alone, educational level, use of calcium and vitamin D supplements, cortisone use, walking/cycling, leisure time physical exercise during the past year, comorbidity, total energy intake, intake of fruits/vegetables and alcohol.

During up to 23 years (mean 18 years) of follow-up and 1,538,627 person-years at risk, 7345 participants (4505 women) experienced a hip fracture. The associations between dietary fatty acid intakes and hip fracture were moderately strong, as shown in Table 2. In the pooled sample, LA intake was inversely associated with hip fracture: In quartile 4, compared with quartile 1, the multivariable-adjusted HR was 0.89 (95% CI 0.81, 0.98) and per SD increase (HR = 0.96 [95% CI 0.93, 0.99]). EPA (HR/SD increase = 1.03 [95% CI 1.01, 1.05]), DPA (HR/SD increase = 1.03 [95% CI 1.01, 1.05]), and DHA (HR/SD increase = 1.03 [95% CI 1.0, 1.05]) were directly related to hip fracture risk in the pooled sample. Tests for non-linearity indicated that the direct associations of EPA, DPA, and DHA and the inverse association of LA with hip fracture were non-linear, as illustrated by the pooled sample results in Fig. 2. The estimates were similar in men and women (data not shown). Low intakes were associated with an increased hip fracture risk for the essential fatty acids LA and ALA, and higher intakes were associated with a lower risk. EPA, DPA, and DHA had a J-shaped association with hip fracture, with the strongest hip fracture associations found with higher intakes of DPA. Myristic acid was linearly but non-significantly associated with hip fracture.

**Table 2:** Multivariable adjusted hazard ratio (HR) and 95% confidence intervals (CI) of hip fracture in quartiles and per SD increase of time-updated intake of dietary fatty acids in men, women and the overall pooled sample.

Men					Women				Pooled			
14:0	Quartile	HR (95% CI)	*P*	Combined *P*	Quartile	HR (95% CI)	*P*	Combined *P*	Quartile	HR (95% CI)	*P*	Combined *P*
	1	1 (Ref.)		0.617	1	1 (Ref.)		0.604	1	1 (Ref.)		0.636
	2	1.01 (0.90, 1.13)	0.885		2	1.01 (0.91, 1.13)	0.806		2	0.99 (0.92, 1.06)	0.705	
	3	0.97 (0.86, 1.09)	0.590		3	0.95 (0.86, 1.05)	0.319		3	0.99 (0.92, 1.07)	0.895	
	4	0.94 (0.82, 1.07)	0.321		4	0.99 (0.88, 1.10)	0.838		4	0.95 (0.87, 1.04)	0.254	
*Per SD*		*0.98 (0.94, 1.02)*	*0.398*			*0.98 (0.94, 1.03)*	*0.511*			*0.98 (0.95, 1.01)*	*0.229*	

18:2 n-6	Quartile	HR (95% CI)	*P*	Combined *P*	Quartile	HR (95% CI)	*P*	Combined *P*	Quartile	HR (95% CI)	*P*	Combined *P*
	1	1 (Ref.)		0.439	1	1 (Ref.)		0.082	1	1 (Ref.)		0.017
	2	0.95 (0.84, 1.06)	0.356		2	0.96 (0.87, 1.05)	0.365		2	0.97 (0.90, 1.04)	0.413	
	3	0.91 (0.80, 1.03)	0.123		3	0.97 (0.87, 1.08)	0.598		3	0.89 (0.81, 0.98)	0.014	
	4	0.91 (0.80, 1.05)	0.206		4	0.87 (0.76, 1.00)	0.046		4	0.89 (0.81, 0.97)	0.013	
*Per SD*		*0.96 (0.91, 1.01)*	*0.153*			*0.96 (0.91, 1.01)*	*0.096*			*0.96 (0.93, 0.99)*	*0.025*	

18:3 n-3	Quartile	HR (95% CI)	*P*	Combined *P*	Quartile	HR (95% CI)	*P*	Combined *P*	Quartile	HR (95% CI)	*P*	Combined *P*
	1	1 (Ref.)		0.362	1	1 (Ref.)		0.370	1	1 (Ref.)		0.110
	2	1.00 (0.89, 1.13)	0.982		2	1.03 (0.94, 1.14)	0.534		2	1.01 (0.94, 1.08)	0.803	
	3	0.94 (0.84, 1.06)	0.341		3	1.08 (0.94, 1.24)	0.261		3	0.96 (0.87, 1.05)	0.370	
	4	0.90 (0.76, 1.05)	0.172		4	1.00 (0.84, 1.19)	0.988		4	0.91 (0.82, 1.01)	0.085	
*Per SD*		*0.97 (0.92, 1.01)*	*0.163*			*0.98 (0.94, 1.03)*	*0.370*			*0.97(0.94, 1.00)*	*0.09*	

20:5 n-3	Quartile	HR (95% CI)	*P*	Combined *P*	Quartile	HR (95% CI)	*P*	Combined *P*	Quartile	HR (95% CI)	*P*	Combined *P*
	1	1 (Ref.)		0.427	1	1 (Ref.)		0.826	1	1 (Ref.)		0.409
	2	0.92 (0.82, 1.04)	0.177		2	1.00 (0.91, 1.11)	0.960		2	0.97 (0.90, 1.05)	0.435	
	3	0.96 (0.85, 1.09)	0.544		3	1.00 (0.91, 1.11)	0.939		3	1.00 (0.92, 1.08)	0.917	
	4	1.01 (0.90, 1.14)	0.865		4	1.04 (0.94, 1.15)	0.438		4	1.03 (0.96, 1.11)	0.369	
*Per SD*		*1.02 (0.99, 1.05)*	*0.159*			*1.03 (0.99, 1.07)*	*0.158*			*1.03 (1.01, 1.05)*	*0.016*	

22:5 n-3	Quartile	HR (95% CI)	*P*	Combined *P*	Quartile	HR (95% CI)	*P*	Combined *P*	Quartile	HR (95% CI)	*P*	Combined *P*
	1	1 (Ref.)		0.816	1	1 (Ref.)		0.607	1	1 (Ref.)		0.058
	2	1.00 (0.88, 1.15)	0.965		2	1.02 (0.93, 1.13)	0.648		2	0.92 (0.86, 1.00)	0.038	
	3	0.98 (0.85, 1.12)	0.738		3	0.99 (0.89, 1.09)	0.762		3	0.97 (0.90, 1.05)	0.458	
	4	1.04 (0.91, 1.18)	0.550		4	1.05 (0.95, 1.16)	0.378		4	1.02 (0.94, 1.11)	0.597	
*Per SD*		*1.02 (0.99, 1.05)*	*0.167*			*1.03 (0.99, 1.07)*	*0.120*			*1.03 (1.01, 1.05)*	*0.014*	

22:6 n-3	Quartile	HR (95% CI)	*P*	Combined *P*	Quartile	HR (95% CI)	*P*	Combined *P*	Quartile	HR (95% CI)	*P*	Combined *P*
	1	1 (Ref.)		0.785	1	1 (Ref.)		0.813	1	1 (Ref.)		0.813
	2	0.97 (0.85, 1.10)	0.628		2	1.02 (0.92, 1.12)	0.736		2	1.02 (0.92, 1.12)	0.736	
	3	0.96 (0.84, 1.08)	0.475		3	1.03 (0.93, 1.14)	0.612		3	1.03 (0.93, 1.14)	0.612	
	4	1.01 (0.89, 1.15)	0.880		4	1.05 (0.95, 1.18)	0.340		4	1.05 (0.95, 1.18)	0.340	
*Per SD*		*1.02 (0.99, 1.05)*	*0.146*			*1.03 (0.99, 1.07)*	*0.181*			*1.03 (1.0, 1.05)*	*0.017*	

14:0 Myristic acid, 18:2 n-6 Linoleic acid, 18:3 n-3 α- linolenic acid, 20:5 n-3 Eicosapentaenoic acid, 22:5 n-3 Docosapentaenoic acid, 22:6 n-3 Docosahexaenoic acid.

### 3.3 Sensitivity analyses

The sensitivity analyses 1 and 2, including dairy products or fish and total meat, did not materially affect the results. However, confidence intervals widened, especially for the association between long chain n-3 fatty acids and hip fracture adjusting for fish and total meat (Supplemental Fig. S1). The third sensitivity analysis, additionally adjusting for protein, calcium and vitamin D gave similar results (data not shown), as did the fourth fully adjusted model, depicted in supplemental Fig. S2. The fifth sensitivity analysis revealed no association between SFA, lauric acid, palmitic acid, and stearic acid and hip fracture (Supplemental Figure S3).

### 3.4 Relative validation study

Characteristics and the residual adjusted intake of fatty acids among participants in the sub-cohorts are shown in Supplemental Table S1. The mean intakes of the dietary fatty acids in grams per day differed between men and women but were consistent, comparing the results from the different investigations in both sexes. Men, in general, had higher absolute intakes of respective fatty acid. Among women, however, the intakes calculated from the 1987 FFQ were lower than those calculated from the two later FFQs. This is consistent with the lower reported average energy intake calculated from the 1987 FFQ and the lowest number of dietary items in this FFQ. Among men, the intakes were more consistent over the three FFQs. The proportions (percentage of total fat) of the different fatty acids in adipose tissue were similar in women and men (Supplemental Table S2). The most abundant fatty acid in adipose tissue was oleic acid (18:1, 51–53 %), and the fatty acid with the lowest proportion was EPA (0.18%). Table 3 presents the correlation coefficients between the adipose tissue fatty acid content, the fatty acid intake calculated from each FFQ, and the long-term average intake of the three FFQs. The strongest correlations were consistently found for LA, ALA, EPA, DHA, and myristic acid, with correlations > 0.25. In women, the correlations were weaker for the correlations between adipose tissue fatty acids and fatty acids calculated from the 1987 FFQ. The partial correlation coefficients were similar to the unadjusted correlations (Table 4).

**Table 3:** Correlation coefficients with 95 % confidence intervals between adipose tissue fatty acids and corresponding fatty acids derived from the food frequency questionnaires (FFQ) administered in 1987, 1997, 2003-09 in sub-cohort 1 (women), and in 1997, 2009 and 2010-11 in participants in the sub-cohort 2 (men), and the mean of all three FFQs.

Sub-cohort 1, women, n = 489 Fatty acid		FFQ 1987	FFQ 1997	FFQ 2003-09	Mean of all three FFQs
14:0	Myristic acid	0.26 (0.16; 0.35)	0.36 (0.28; 0.45)	0.44 (0.36; 0.51)	0.48 (0.40; 0.55)
16:0	Palmitic acid	0.14 (0.05; 0.22)	0.13 (0.03; 0.22)	0.25 (0.15; 0.34)	0.24 (0.15; 0.34)
18:0	Stearic acid	0.10 (−0.0001; 0.20)	0.11 (0.03; 0.20)	0.11 (0.02; 0.19)	0.15 (0.06; 0.24)
16:1	Palmitoleic acid	0.08 (−0.02; 0.17)	0.08 (-0.01; 0.18)	0.11 (0.02; 0.21)	0.13 (0.03; 0.22)
18:1	Oleic acid	0.08 (−0.002; 0.17)	0.13 (0.04; 0.22)	0.23 (0.15; 0.32)	0.21 (0.13; 0.31)
18:2 n-6	Linoleic acid, LA	0.18 (0.09; 0.28)	0.22 (0.12; 0.32)	0.41 (0.31; 0.51)	0.38 (0.29; 0.48)
20:4 n-6	Arachidonic acid	0.12 (0.03; 0.20)	0.12 (0.05; 0.20)	0.10 (0.01; 0.18)	0.15 (0.07; 0.24)
18:3 n-3	α- linolenic acid, ALA	0.19 (0.10; 0.28)	0.17 (0.08; 0.26)	0.36 (0.25; 0.47)	0.37 (0.28; 0.46)
20:5 n-3	Eicosapentaenoic acid, EPA	0.17 (0.08; 0.26)	0.22 (0.14; 0.31)	0.32 (0.24; 0.40)	0.31 (0.22; 0.39)
22:5 n-3	Docosapentaenoic acid, DPA	0.10 (0.008; 0.20)	0.21 (0.12; 0.30)	0.24 (0.15; 0.32)	0.25 (0.16; 0.34)
22:6 n-3	Docosahexaenoic acid, DHA	0.21 (0.12; 0.30)	0.29 (0.20; 0.37)	0.41 (0.34; 0.49)	0.41 (0.33; 0.49)

Sub-cohort 2, men, n = 495 Fatty acid		FFQ 1997	FFQ 2009	FFQ 2010-11	Mean of all three FFQs
14:0	Myristic acid	0.26 (0.18; 0.35)	0.28 (0.21; 0.35)	0.37 (0.29; 0.46)	0.35 (0.27; 0.42)
16:0	Palmitic acid	0.14 (0.05; 0.22)	0.12 (0.04; 0.21)	0.15 (0.06; 0.24)	0.17 (0.08; 0.26)
18:0	Stearic acid	0.04 (−0.05; 0.13)	0.18 (0.09; 0.26)	0.18 (0.10; 0.28)	0.13 (0.03; 0.22)
16:1	Palmitoleic acid	0.05 (−0.04; 0.14)	−0.10 (−0.18; −0.03)	−0.006 (−0.09; 0.08)	−0.06 (−0.14; 0.03)
18:1	Oleic acid	0.08 (−0.007; 0.18)	0.17 (0.09; 0.26)	−0.02 (−0.11; 0.07)	0.17 (0.08; 0.25)
18:2 n-6	Linoleic acid, LA	0.29 (0.20; 0.37)	0.42 (0.34; 0.50)	0.34 (0.25; 0.42)	0.45 (0.38; 0.52)
20:4 n-6	Arachidonic acid	0.10 (0.02; 0.18)	0.05 (−0.03; 0.14)	0.06 (−0.02; 0.14)	0.08 (−0.004; 0.16)
18:3 n-3	α- linolenic acid, ALA	0.29 (0.20; 0.37)	0.25 (0.17; 0.34)	0.29 (0.21; 0.38)	0.30 (0.22; 0.39)
20:5 n-3	Eicosapentaenoic acid, EPA	0.32 (0.24; 0.40)	0.38 (0.30; 0.46)	0.38 (0.30; 0.45)	0.42 (0.34; 0.49)
22:5 n-3	Docosapentaenoic acid, DPA	0.14 (0.05; 0.23)	0.21 (0.12; 0.30)	0.24 (0.16; 0.33)	0.23 (0.14; 0.31)
22:6 n-3	Docosahexaenoic acid, DHA	0.32 (0.24; 0.41)	0.40 (0.31; 0.48)	0.40 (0.32; 0.48)	0.42 (0.34; 0.50)

n-3 fatty acids with Spearman and the other fatty acids with Pearson correlation analysis.

**Table 4:** Partial correlations between fatty acids in adipose tissue and those derived from FFQ2003-09 in sub-cohort 1 (women) and from FFQ2010-11 in sub-cohort 2 (men).

								n-6		n-3			
			14:0	16:0	18:0	16:1	18:1	18:2	20:4	18:3	20:5	22:5	22:6
Sub-cohort 1	Model 1	n=484	0.41^3^	0.25^3^	0.12^2^	0.11^1^	0.22^3^	0.41^3^	0.06	0.36^3^	0.30^3^	0.25^3^	0.45^3^
	Model 2	n=235	0.44^3^	0.28^3^	0.12	0.12	0.17^1^	0.46^3^	0.13^1^	0.37^3^	0.25^3^	0.25^3^	0.45^3^

Sub-cohort 2	Model 1	n=474	0.36^3^	0.17^3^	0.21^2^	−0.02	0.0007	0.34^3^	0.06	0.31^3^	0.34^3^	0.24^3^	0.39^3^

Sub-cohort 1: Model 1: Adjusted for age at participation, body mass index (kg/m^2^), energy intake, Model 2: Adjusted for age at participation, body mass index (kg/m^2^) and energy intake and physical activity level.

Sub-cohort 2: Model 1: Adjusted for age at participation, body mass index (kg/m^2^), energy intake and exercise level during the past month.

^1^p<0.05, ^2^p<0.01, ^3^p<0.001.

## 4 Discussion

The results of the present study show that individual dietary fatty acids relate to hip fracture risk over 23 years. A low to moderate intake of LA and high intakes of EPA, DPA, and DHA were independently associated with higher hip fracture risk, all showing non-linear associations. The analysis also revealed that ALA and myristic acid were linearly and inversely associated with hip fracture risk, although in a non-significant way. The results were independent of several lifestyle factors, comorbidity, and intakes of vegetables, fruits, and dairy products. The study confirmed the validity of the FFQs to capture the intake of the specific long-chain dietary fatty acids: EPA, DPA, and DHA in women [22], and the present results further extend the validity to men. The present study also established the validity of the FFQ-reported dietary intakes of LA, ALA, and myristic acid.

The epidemiological evidence for the association between dietary fatty acid intakes and hip fracture risk has been uncertain and conflicting [9,[15], [16], [17], [18],^37^], and the results of the present study add to the body of evidence of a true association. That the present study detected associations between individual polyunsaturated fatty acid acids and hip fracture risk, in contracts to the results in [17], may be attributable to a wider exposure range and that the number of hip fractures was high. Hip fracture cases were also ascertained by linkage to national registers, which prevents selective loss to follow-up. The causality of the present study's findings that higher intakes of LA (ALA although non-significantly), but not long-chain n-3 fatty acids, were modestly and inversely associated with hip fracture is supported by the results in a Mendelian randomization (MR) study [38]. Genetic predisposition of higher plasma levels of ALA and LA were associated with higher estimated BMD. In comparison, higher plasma levels of EPA and DPA were associated with lower BMD, which translated to lower odds of fracture for ALA and LA and higher odds of fracture for EPA and DPA [38]. Still, fatty acid intake is difficult to capture in a dietary study due to reporting biases [19] and may also reflect other dietary components than fatty acids due to co-existence in foods. This makes it difficult to quantify and interpret individual fatty acids’ potential biological effects on the pathological process of hip fracture development, even if no residual confounding would exist. For example fish and shellfish provides vitamin D, protein and selenium in addition to long chain-3 fatty acids [39].

Biomarker fatty acids measured in plasma and adipose tissue mirror dietary intake of fatty acids to a varying degree [20]. Valid estimates of the intake of the estimated individual fatty acids are important for the investigated associations between individual fatty acids and hip fracture risk. Our relative validation study, using biomarker fatty acids measured in adipose tissue reflective of long-term dietary fatty acid intake [40], indicated that the FFQs capture the estimated dietary intake of myristic acid, ALA, LA, EPA, DPA, and DHA (r > 0.25) to a satisfactory degree. The fatty acids measured in adipose tissue correlated with those estimated by the FFQs administered years before the sample collection, indicating long-term reliability. The correlations were the weakest for the dietary fatty acids estimated in the earliest FFQ in women (1987) and the strongest for those estimated in the FFQs used close to the clinical sub-cohorts, i.e., at the time of sample collection. The FFQs cover the diet over the last year, and the adipose tissue fatty acids reflect the dietary fat quality in the previous years [40]. In addition to more than a decade’s difference in time intervals between FFQs and the fat biopsy, the different strengths of the correlations are influenced by changes in food habits over time and the fatty acid content of the dietary fat used in dishes in the food database. Compared with previous studies from the United States, the strength of the correlations in our study was more robust for the n3 fatty acids but weaker for LA [41,42]. Different studies reported varying strengths of the correlations may also be influenced by different dietary assessment methods. Myristic acid in adipose tissue has previously been found to correlate with dairy fat [43].

Long-chain fatty acids, such as LA, ALA, EPA, and DHA, are central structural components of cell membranes and impact membrane fluidity and permeability, activity of membrane-bound enzymes and receptors, and signal transduction [44]. Long-chain fatty acids are also precursors of a range of metabolites called eicosanoids and docosanoids. These metabolites include prostaglandins, leukotrienes, thromboxanes, lipoxins, and resolvins, which may affect bone metabolism in several ways. For example, these metabolites modulate bone metabolism by induction of inflammation and oxidative stress [45]. Fatty acids can also bind to specific receptors in the cell membrane or nucleus of osteoblasts and osteoclasts, which in turn induces signals that lead to gene transcription and protein synthesis, influencing the growth and function of the tissue [46]. Experimental studies have suggested that PUFA inhibits osteoclast activity and enhances osteoblasts' activity, while saturated fatty acids are detrimental to osteoblasts and may stimulate osteoclastogenesis [47,48]. Consistent with this, a smaller randomized cross-over feeding trial showed that a diet high in plant-based n-6 (LA) or n-3 (ALA) fatty acids from walnuts and flaxseed oil, compared to a Western-type diet higher in saturated fatty acids, decreased expression of a bone resorption marker (serum N-telopeptides [NTx]) [49]. A higher n-3 content had the most significant effect. NTx further correlated with the pro-inflammatory cytokine tumor necrosis factor alpha (TNF-α), suggesting an anti-inflammatory effect of a higher ALA intake. Additionally, the different diets did not affect a marker of bone formation (bone-specific alkaline phosphatase) [49]. More recently, a study using a plasma high-resolution metabolomics technique identified mainly LA and metabolites in relation to adverse bone health and lower BMD [50]. These results are not entirely consistent with our results. This disagreement may be because the metabolome is reflective of metabolites at a one-time point (and not per se to dietary LA) and because the risk of hip fracture is related to a low BMD and other age-related pathological processes. Nevertheless, delta-desaturases and elongases endogenously metabolize LA to dihomo-γ-linoleic acid (20:3 n-6) and AA (20:4 n-6). Both dihomo-γ-linoleic and AA are precursors of eicosanoids, including prostaglandins and leukotrienes. Some of these are involved in inflammation, while others, such as lipoxins and resolvins, are anti-inflammatory [51]. Previous studies using serum biomarker fatty acids have established LA to be associated with cardio-protection [52] and a decreased risk of diabetes [53]. At the same time, dihomo-γ-linoleic acid is associated with an increased risk of cardiovascular mortality [54]. It should also be noted that ALA is converted to EPA by the same enzymes [51] and further to other prostaglandins.

That higher intake of EPA and DHA associated with an increased risk of hip fracture in the present study may be due to a relatively low physiological demand for these fatty acids or that the intake in the study population was relatively low. The Nordic Nutrition Recommendations 2023 states that total intake of n-3 fatty acids should contribute to around 1 percent of energy and that ALA intake should account for at least half of this [8]. However, according to the assessment of the European Food Safety Authority there appears to be no safety concern in adults with supplemental intakes of EPA and DHA combined at daily doses up to 5 grams and of EPA alone up to 1.8 grams [55]. In agreement with our findings reported the study by Orchard et al., performed in the Women’s Health Initiative, a higher risk of total fractures with higher consumption of long-chain n-3 fatty acids. The authors argued that any benefit of these fatty acids might require a threshold of intake not reached by their study participants [15]. Thus, our findings warrant further investigation since we found linear adverse associations at higher intakes (although few people achieved such a high intake). The predominant PUFA in the diet is LA, followed by ALA, and the intakes of EPA, DPA, and DHA are several-fold lower [39].

Our present observational and previous MR results [33] are partly supported by results of randomized controlled trials (RCT). A systematic review and meta-analysis of RCTs (>6 months) found limited evidence for positive effects of supplemental intake of n-3 and mixed PUFA on bone and skeletal muscle health outcomes but concluded that studies investigating the impact of n-6 fatty acids mainly were lacking [56]. The results of the DO-HEALTH RCT over three years conducted in adults aged 70 years or older did not result in any significant treatment effects of n-3 fatty acids (EPA and DHA) on either incidence of non-vertebral fractures or physical performance [57]. Effects on fracture rates, however, may need a more extended induction period.

Our study has both strengths and weaknesses. Foremost, the two population-based studies used for the present analyses include a large number of hip fractures and involve both men and women. Compared to previous studies using self-reported hip fracture cases, we achieved complete ascertainment of hip fractures using nationwide patient registers with no loss to follow-up. The large sample size ensured a wide exposure range, which enabled the analysis of individual fatty acids. The study further updated the intake of fatty acids twice during follow-up, which is a significant strength. We included several important covariates, including sociodemographic characteristics, intake of fruits and vegetables, alcohol and dairy products, comorbidity, and BMI and the results were robust across several sensitivity analyses including additional dietary covariates. However, there is still a risk of residual or unmeasured confounding. Intake of fruits and vegetables is also a marker of an overall healthy dietary pattern [58], thus including this as a covariate will ensure adjustment for other health characteristics not directly measured in the study. Further, the collection of dietary data is prone to limitations. However, the study questionnaires can rank participants, and the extensive study size will compensate for random misclassification [59]. Further, the relative validation of the FFQs by using fatty acids measured in adipose tissue strengthens the validity of the estimated intake of dietary fatty acids and, thus, our study exposures. Fatty acid intake may also reflect other dietary components due to co-existence in foods, for example fish, meat and dairy products.

In conclusion, a low to moderate intake of LA and higher intakes of EPA, DPA, and DHA were associated with an increased risk of hip fracture in both men and women. Furthermore, a higher intake of LA was associated modestly and inversely with hip fracture risk, consistent with dietary guidelines. The present results indicate that dietary fatty acid composition is essential for the optimal prevention of fragility fractures. The results should be confirmed in other study populations.

## Funding

The study was supported by funding from the Swedish Research Council(https://www.vr.se; grants No. 2015-03257,2017-00644,2017-06100, and2019-01291to Karl Michaëlsson) and funding from Olle Engkvist Byggmästares stiftelse (SOEB). We acknowledge the national research infrastructure (SIMPLER) for data generation, availability, computational facilities, and resources. SIMPLER receives funding through the Swedish Research Council under grant No. 2017-00644 and 2021-00160(to Uppsala University and Karl Michaëlsson). The sponsor was not involved in the study design, data collection, statistical analysis, interpretation of results, report writing, or publication restrictions.

## Ethical statement

The study complied with the Declaration of Helsinki, and the regional ethics committees at Uppsala University, Uppsala, and Karolinska Institutet, Stockholm, Sweden, approved the study. All study participants provided informed consent to participate in the studies.

## Conflict of interests

The authors declare that they have no known competing financial interests or personal relationships that could have appeared to influence the work reported in this paper.
