# Supplementary material for: Dietary fatty acids and incident hip fractures in cohorts of women and men. A relative validation and follow-up study
Source: J Nutr Health Aging. 2024 Apr 25;28(7):100247. doi: 10.1016/j.jnha.2024.100247 (PMC12433805; doi:10.1016/j.jnha.2024.100247)
Supplement: Supplementary file 1 [file mmc1.docx]

Supplemental material

**Dietary fatty acids and incident hip fractures in cohorts of women and men. A relative validation and follow-up study**

Eva Warensjö Lemming, Liisa Byberg, Jonas Höijer, Susanna C. Larsson, Alicja Wolk and Karl Michaëlsson

**Supplemental Table 1**. Characteristics among those with measured adipose fatty acids at the time of the investigations 1987, 1997 and 2003-09 in women and 1997, 2009 and 2010-11 in men.

|  | Women (n= 489) | | | | | | Men (n=495) | | | | | |
| --- | --- | --- | --- | --- | --- | --- | --- | --- | --- | --- | --- | --- |
|  | 1987 |  | 1997 |  | 2003-09 |  | 1997 |  | 2009 |  | 2010-11 |  |
| Age | 50 (6) |  | 58 (6) |  | 66 (6) |  | 71(3) |  | 83(3) |  | 84 (3) |  |
| Energy intake (kcal) | 1586 (419) |  | 1751 (531) |  | 1700 (518) |  | 2440 (674) |  | 2480 (775) |  | 2480 (721) |  |
| Fat (g/ day) | 49.4 (16.3) |  | 61.4 (23.5) |  | 58.5 (21.0) |  | 82 (29) |  | 84 (31) |  | 87 (31) |  |
| Fat (E%) | 31.0 ( 5.1) |  | 34.7 (6.0) |  | 34.3 (6.1) |  | 30.0 (5.0) |  | 30.3 (4.9) |  | 31.3 (4.7) |  |
| Intake of residual adjusted fatty acids in gram per day | | | | | | | | | | | | |
| 14:0 | 2.5 (1.1) |  | 3.4 (1.7) |  | 3.0 (1.5) |  | 4.7 (2.1) |  | 4.3 (2.0) |  | 4.6 (2.1) |  |
| 16:0 | 10.8 (3.8) |  | 13.9 (5.6) |  | 12.9 (5.0) |  | 18.7 (6.7) |  | 17.9 (7.1) |  | 18.8 (7.3) |  |
| 18:0 | 4.9 (1.8) |  | 5.7 (2.3) |  | 5.3 (2.0) |  | 7.5 (2.7) |  | 7.8 (3.2) |  | 8.3(3.2) |  |
| 16:1 | 0.96 (0.3) |  | 1.1(0.4) |  | 1.1(0.4) |  | 1.4 (0.5) |  | 1.3 (0.6) |  | 1.3 (0.5) |  |
| 18:1 | 14.5 (4.9) |  | 18.4 (7.0) |  | 18.1(6.6) |  | 24.6 (8.5) |  | 25.1 (9.5) |  | 26.1 (9.1) |  |
| 18:2 n-6 | 5.8 (2.1) |  | 6.5 (2.9) |  | 6.2 (2.3) |  | 8.0 (2.9) |  | 9.2 (4.1) |  | 9.3 (3.8) |  |
| 18:3 n-3 | 0.82 (0.30) |  | 1.2 (0.50) |  | 1.3 (0.5) |  | 1.5 (0.6) |  | 2.1 (1.3) |  | 2.2 (1.3) |  |
| 20:4 n-6 | 0.09 (0.04) |  | 0.09 (0.04) |  | 0.10 (0.04) |  | 0.13 (0.05) |  | 0.12 (0.08) |  | 0.11 (0.06) |  |
| 20:5 n-3 | 0.07 (0.04) |  | 0.12 (0.09) |  | 0.14 (0.10) |  | 0.15 (0.13) |  | 0.18 (0.16) |  | 0.16 (0.11) |  |
| 22:5 n-3 | 0.03 (0.01) |  | 0.04 (0.03) |  | 0.05 (0.03) |  | 0.06 (0.04) |  | 0.07 (0.06) |  | 0.07 (0.04) |  |
| 22:6 n-3 | 0.14 (0.08) |  | 0.24 (0.19) |  | 0.28 (0.18) |  | 0.31 (0.27) |  | 0.45 (0.37) |  | 0.41 (0.26) |  |

E Energy, E% percent of energy

14:0 Myristic acid, 16:0 Palmitic acid, 18:0 Stearic acid, 16:1 Palmitoleic acid, 18:1 Oleic acid, 18:2 n-6 Linoleic acid, 18:3 n-3 α- linolenic acid, 20:4 n-6 Arachidonic acid, 20:5 n-3 Eicosapentaenoic acid, 22:5 n-3 Docosapentaenoic acid, 22:6 n-3 Docosahexaenoic acid

**Supplemental Table 2**. Proportions of adipose tissue fatty acids among participants in the two clinical sub-cohorts.

|  |  | Women (n=489) | Men (n=495) |
| --- | --- | --- | --- |
|  |  | % ± SD of total fatty acids | |
| 14:0 | Myristic acid | 2.7 ± 0.51 | 3.3 ± 0.62 |
| 16:0 | Palmitic acid | 19.8 ± 2.1 | 21.5 ± 2.1 |
| 18:0 | Stearic acid | 2.5 ± 0.74 | 3.1 ± 0.78 |
| 16:1 | Palmitoleic acid | 8.3 ± 1.6 | 7.1 ± 1.8 |
| 18:1 | Oleic acid | 53.4 ± 1.9 | 51.3 ± 2.0 |
| 18:2 n-6 | Linoleic acid | 10.0 ± 1.5 | 10.2 ± 1.6 |
| 20:4 n-6 | Arachidonic acid | 0.42 ± 0.10 | 0.40 ± 0.10 |
| 18:3 n-3 | Linolenic acid | 1.1 ± 0.21 | 1.2 ± 0.25 |
| 20:5 n-3 | Eicosapentaenoic acid | 0.18 ± 0.06 | 0.18 ± 0.05 |
| 22:5 n-3 | Docosapentaenoic acid | 0.39 ± 0.11 | 0.35 ± 0.09 |
| 22:6 n-3 | Docosahexaenoic acid | 0.40 ± 0.14 | 0.34 ± 0.13 |

**Supplemental figure 1.**

| Sensitivity analysis 1. Additionally adjusted for dairy products | Sensitivity analysis 2. Additionally adjusted for total meat and fish |
| --- | --- |
| 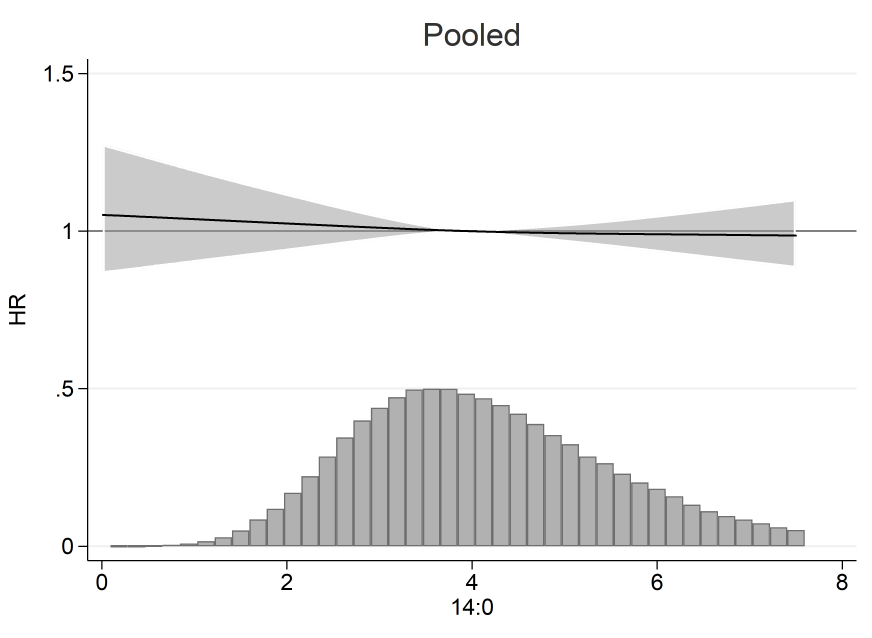 | 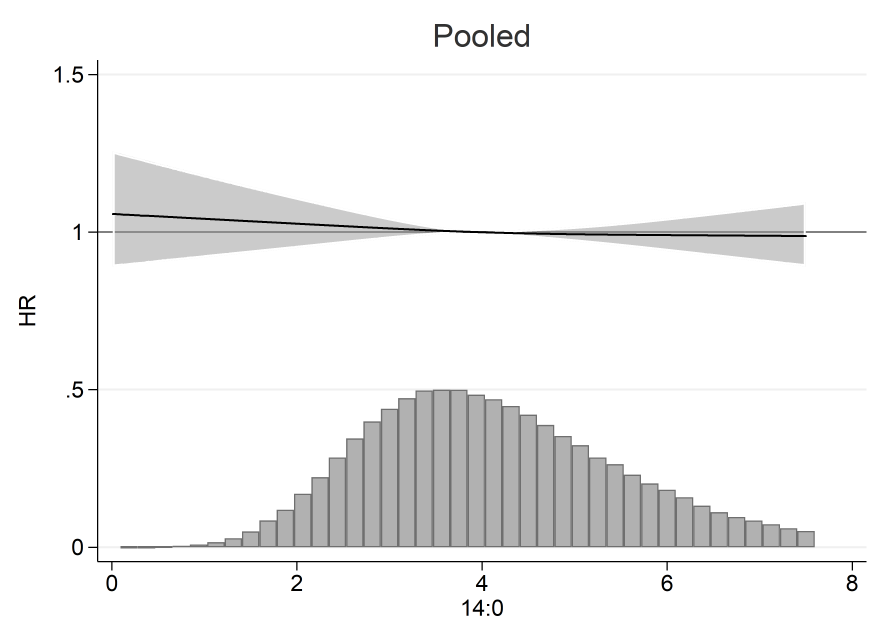 |
| Myristic Acid (14:0) | |
| 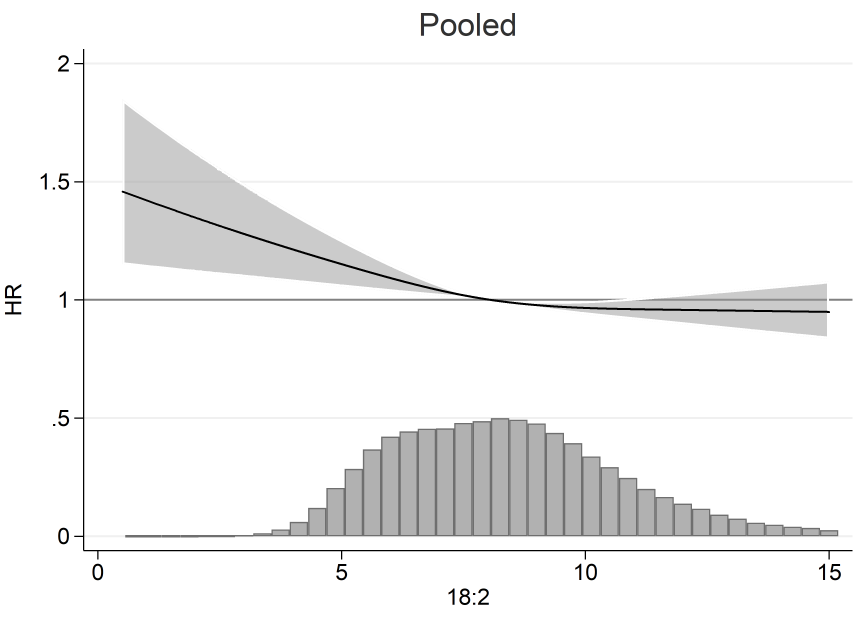 | 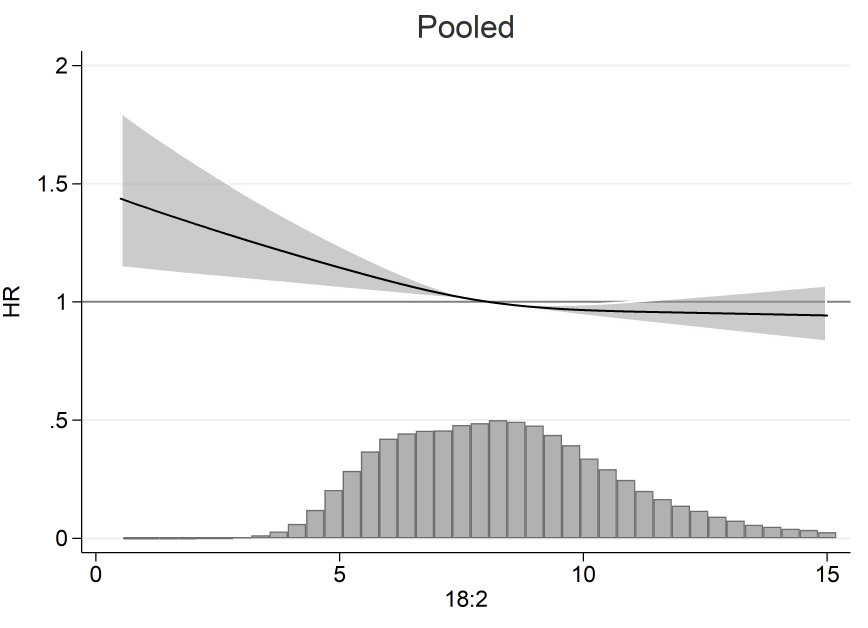 |
| Linoleic Acid (18:2, n-6) | |
| 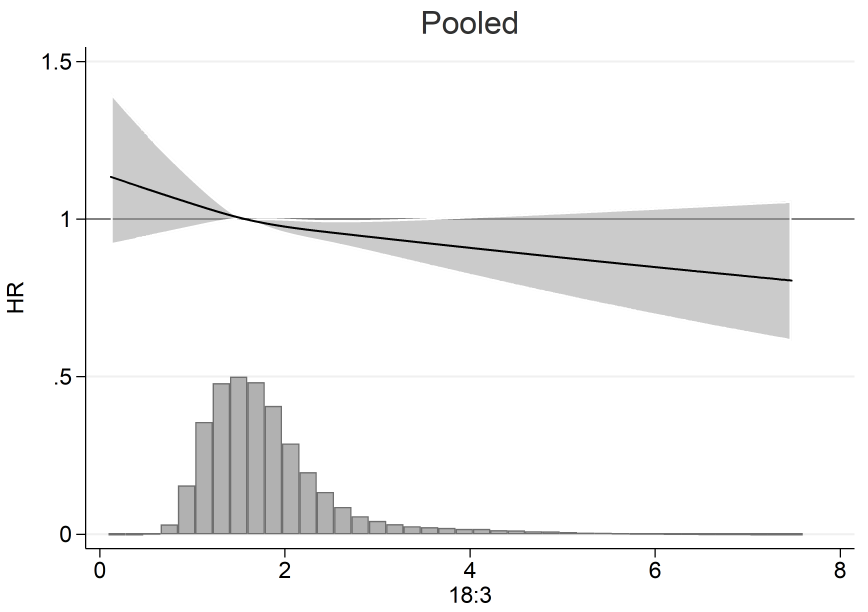 | 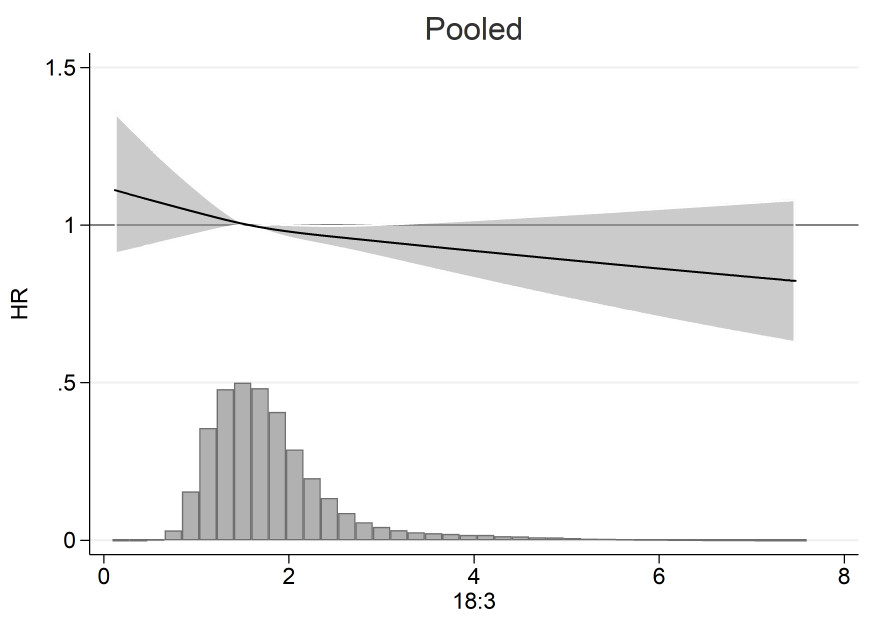 |
| α-Linolenic Acid (18:3 n-3) | |
| 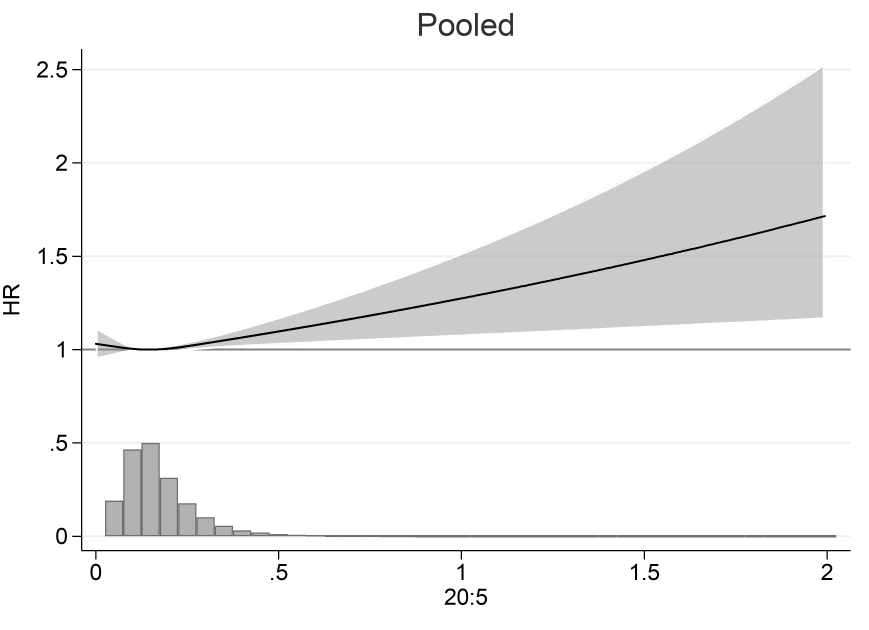 | 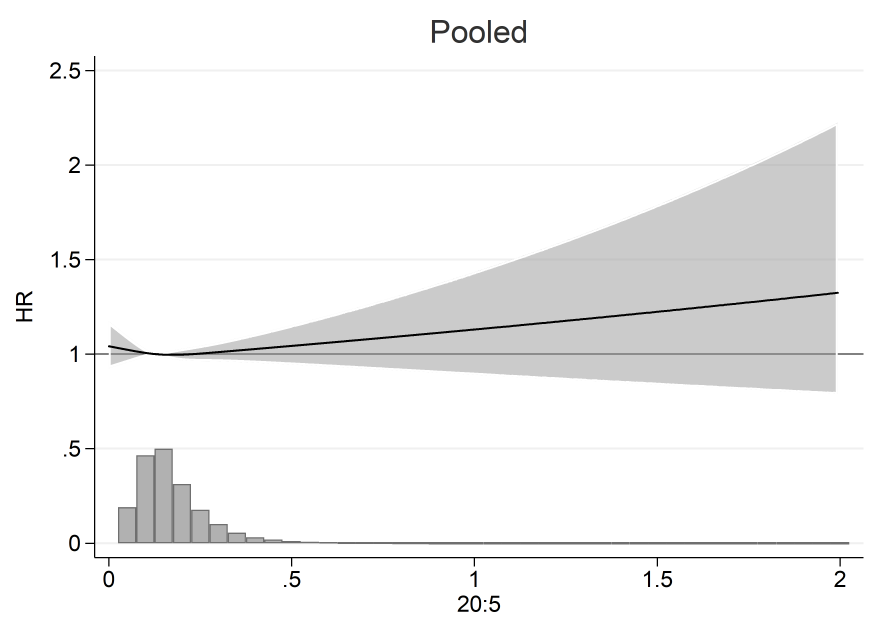 |
| Eicosapentaenoic acid (20:5 n-3) | |
| 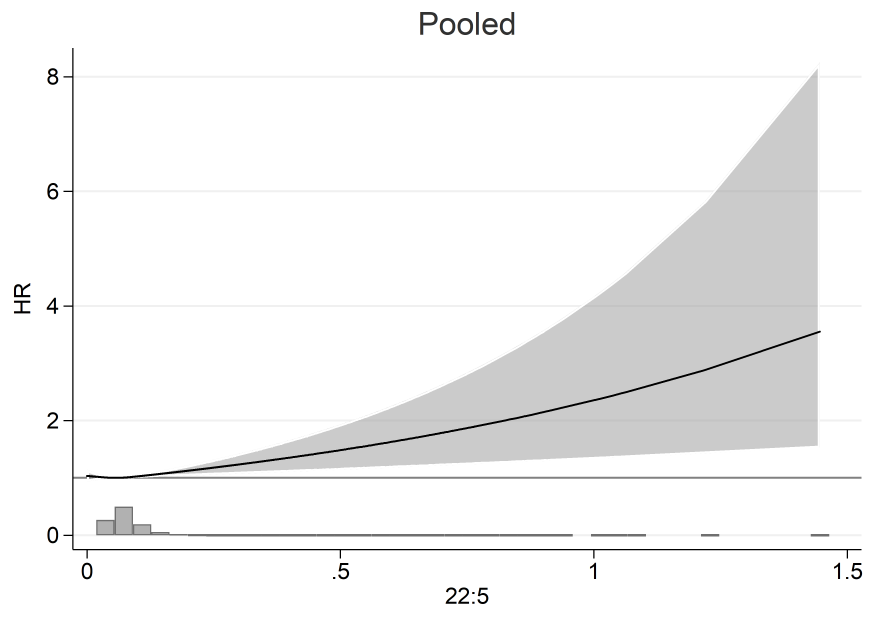 | 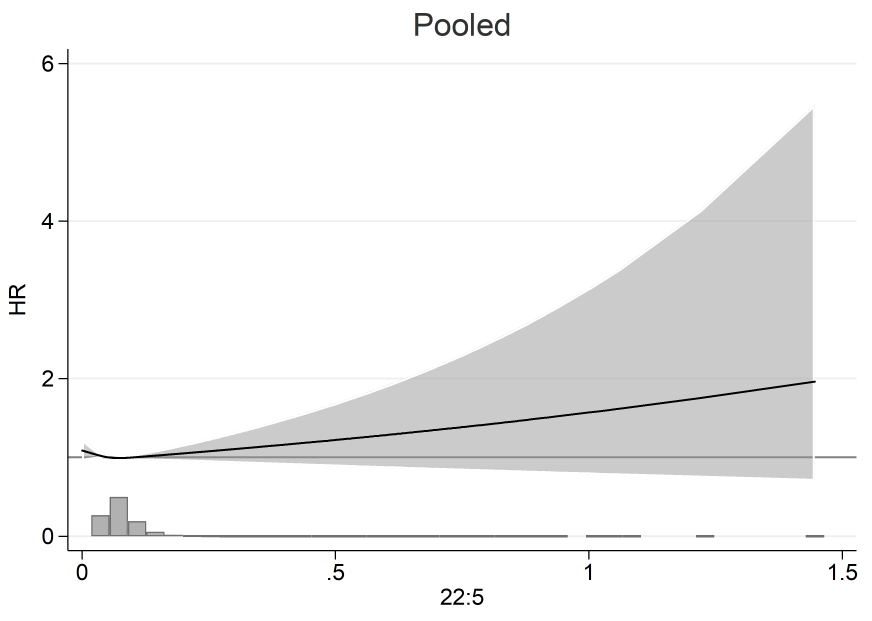 |
| Docosapentaenoic acid (22:5 n-3) | |
| 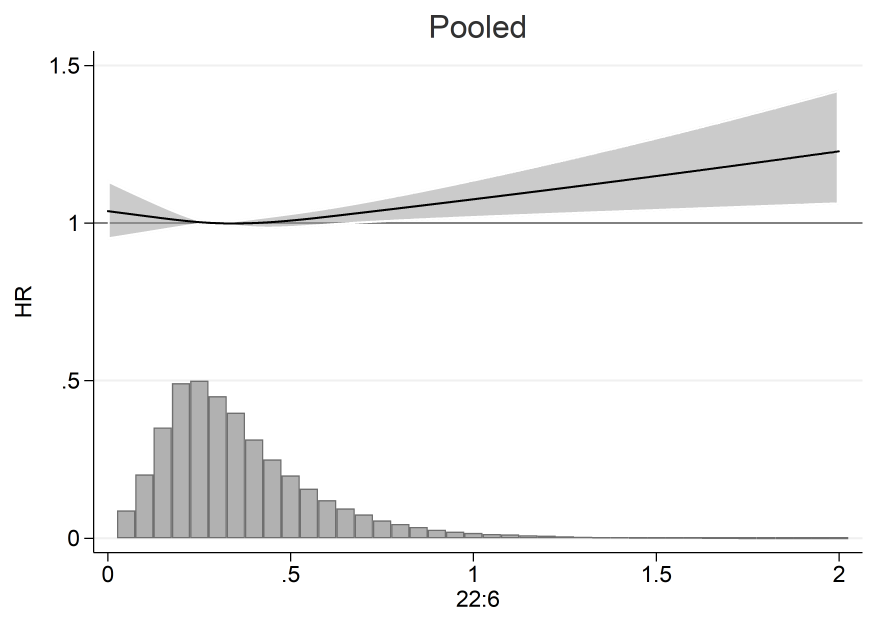 | 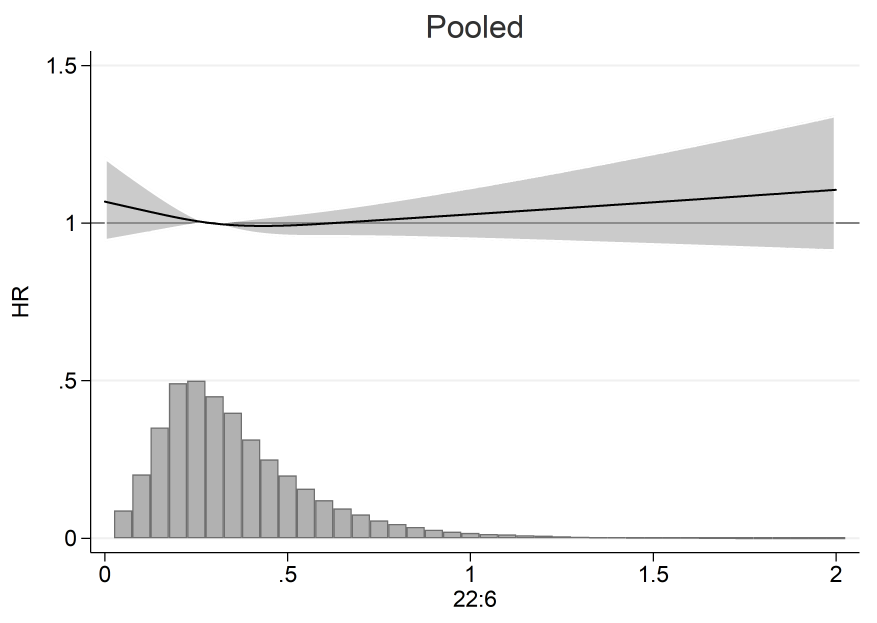 |
| Docosahexaenoic acid (22:6 n-3) | |

The figure shows the results from sensitivity analysis 1 and 2. Depicted are the multivariable adjusted Hazard Ratio (HR, black line) and 95% Confidence Intervals (CI, shaded area) of hip fracture using restricted cubic-splines of time-updated dietary fatty acids in the overall pooled sample. The median intake level of respective fatty acid was used as the reference level in the figure. Depicted below the curve is the distribution of the dietary fatty acid intake among the participants. The intake levels are depicted as the residual adjusted intake in grams per day (x-axis). The multivariable model adjusted for age, height, Body Mass Index, smoking status, living alone, educational level, use of calcium and vitamin D supplements, cortisone use, walking/cycling, leisure time physical exercise during the past year, comorbidity, total energy intake, intake of fruits/vegetables and alcohol. Sensitivity analysis 1 additionally included dairy products and sensitivity analysis 2 included fish and total meat.

**Supplemental figure 2.**

| 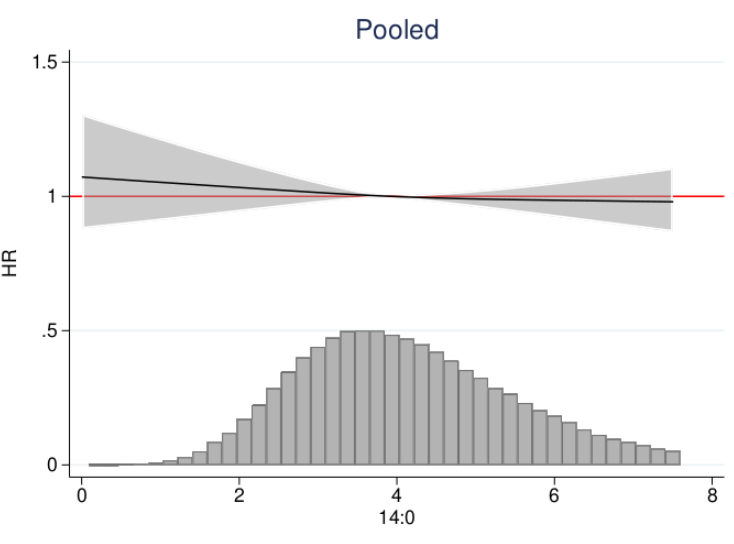 | 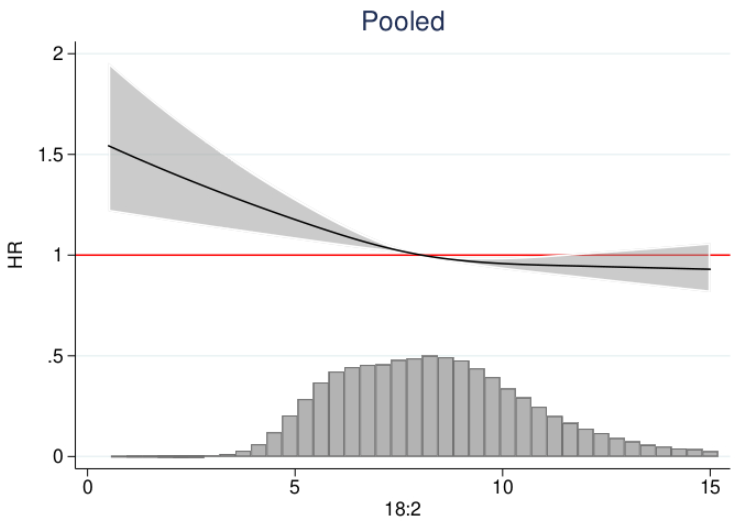 |
| --- | --- |
| Myristic Acid (14:0) | Linoleic Acid (18:2, n-6) |
| 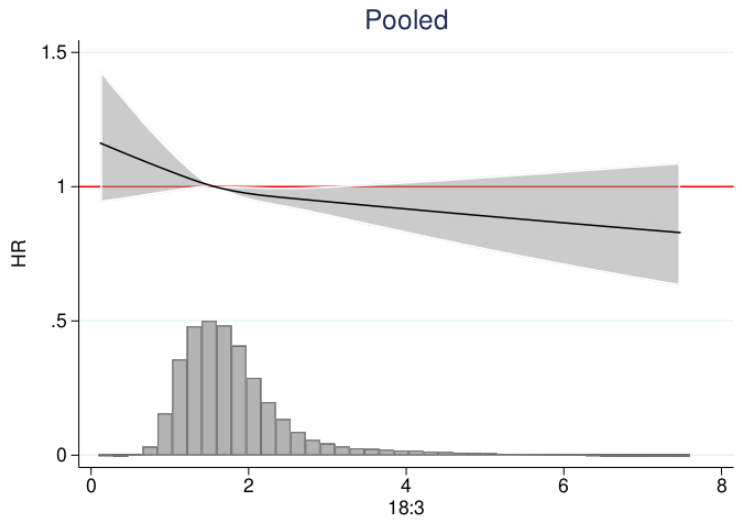 | 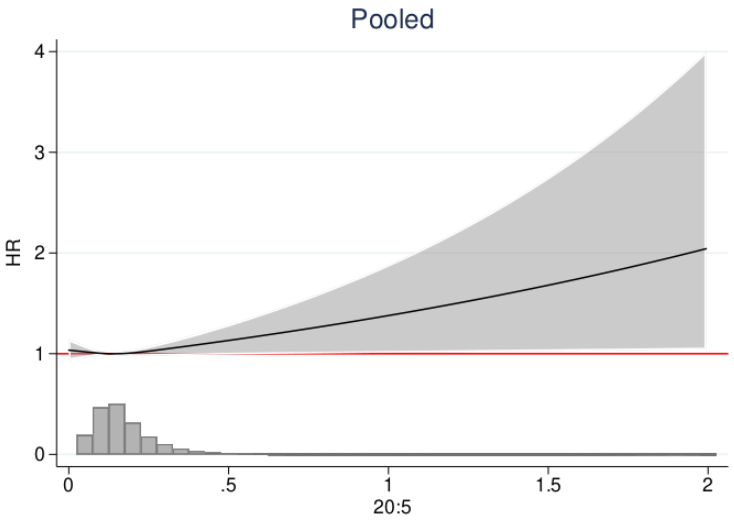 |
| α-Linolenic Acid (18:3 n-3) | Eicosapentaenoic acid (20:5 n-3) |
| 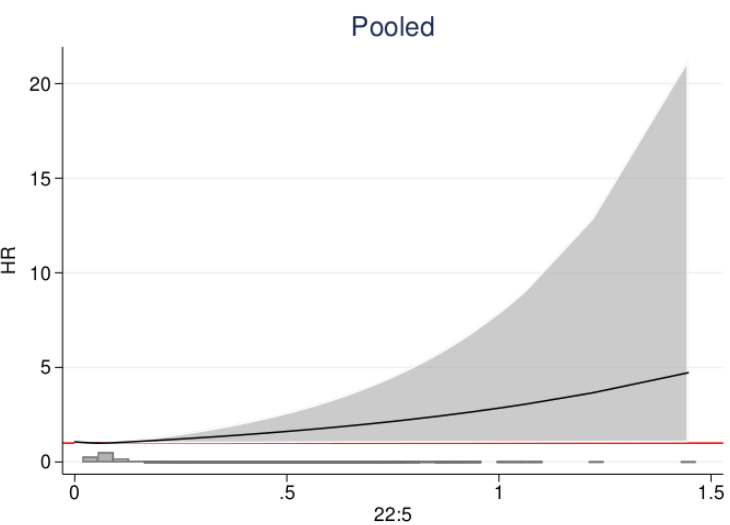 | 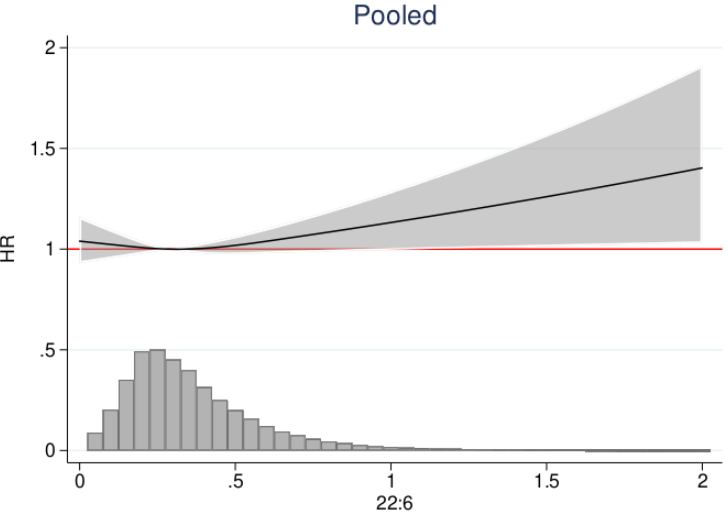 |
| Docosapentaenoic acid (22:5 n-3) | Docosahexaenoic acid (22:6 n-3) |

The figure shows the results from sensitivity analysis 3. Depicted are the multivariable adjusted Hazard Ratio (HR) and 95% Confidence Intervals (CI) of hip fracture using restricted cubic-splines and time-updated specific dietary fatty acids variables in the overall pooled sample. The fatty acids were residual adjusted. Depicted below the curve is the distribution of the dietary fatty acid intake among the participants. The intake levels are depicted as the residual adjusted intake in grams per day (x-axis). The median intake levels of respective fatty acid was used as the reference. The fully adjusted multivariable model was adjusted for age, height, Body Mass Index, smoking status, living alone, educational level, use of calcium and vitamin D supplements, cortisone use, walking/cycling, leisure time physical exercise during the past year, comorbidity, total energy intake, intake of fruits/vegetables, alcohol, dairy products, fish and meat, protein, calcium and vitamin D.

**Supplemental figure 3.**

| 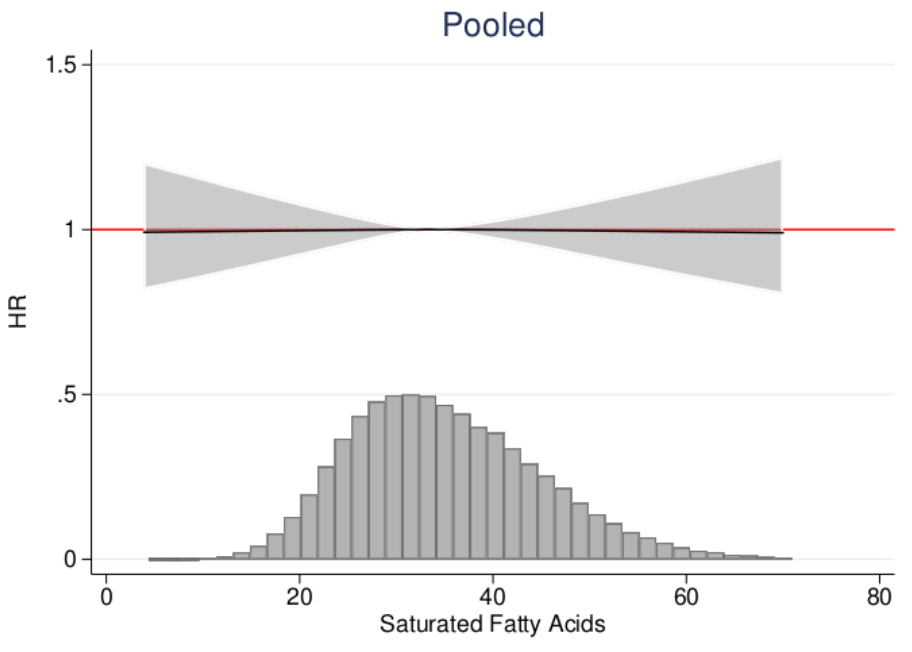  Total saturated fatty acids | 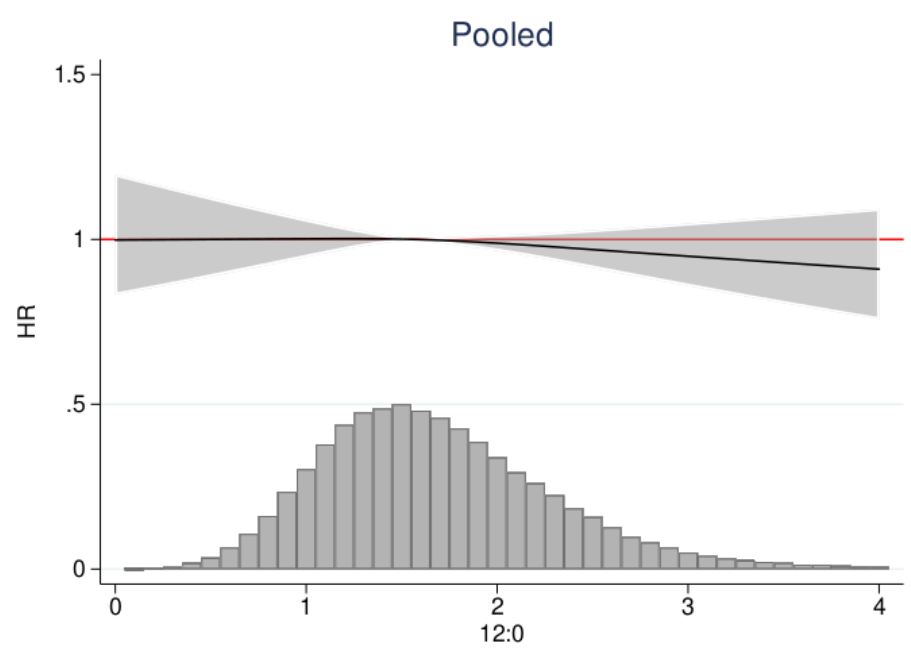  Lauric acid (12:0) |
| --- | --- |
| 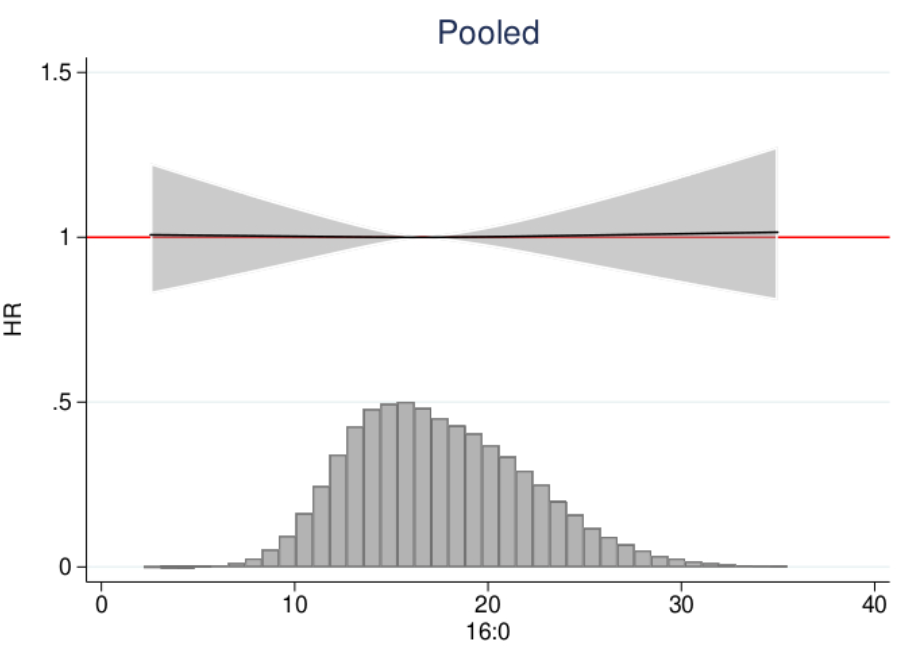  Palmitic acid (16:0) | 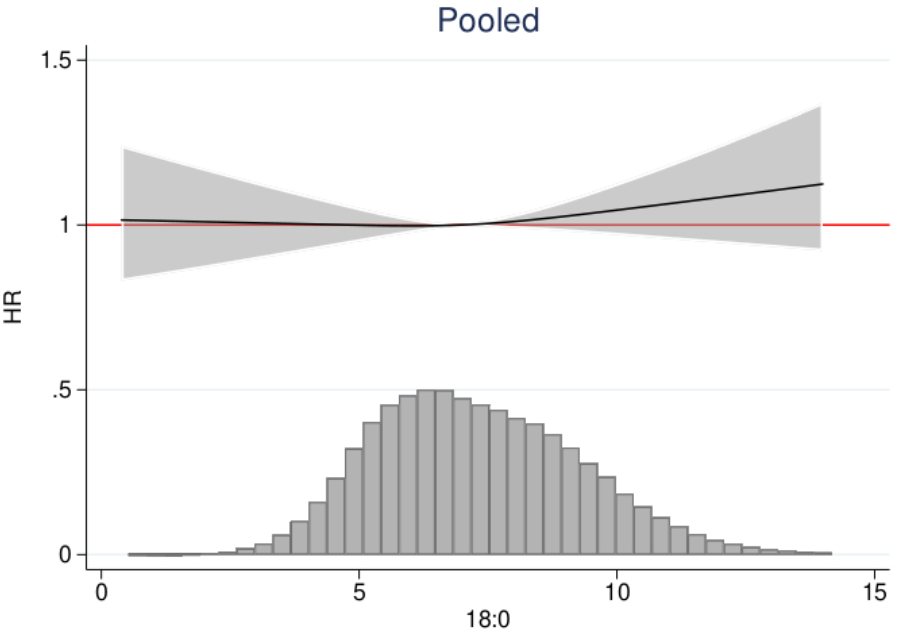  Stearic acid (18:0) |

The figure shows the results from sensitivity analysis 4. Depicted are the multivariable adjusted Hazard Ratio (HR) and 95% Confidence Intervals (CI) of hip fracture using restricted cubic-splines and time-updated specific dietary fatty acids variables in the overall pooled sample. The fatty acids were residual adjusted. Depicted below the curve is the distribution of the dietary fatty acid intake among the participants. The intake levels are depicted as the residual adjusted intake in grams per day (x-axis). The median intake levels of respective fatty acid was used as the reference. The multivariable model was adjusted for age, height, Body Mass Index, smoking status, living alone, educational level, use of calcium and vitamin D supplements, cortisone use, walking/cycling, leisure time physical exercise during the past year, comorbidity, total energy intake, intake of fruits/vegetables, alcohol and dairy products.
